# The Roles of Entropy and Kinetics in Structure Prediction

**DOI:** 10.1371/journal.pone.0005840

**Published:** 2009-06-09

**Authors:** Gregory R. Bowman, Vijay S. Pande

**Affiliations:** 1 Biophysics Program, Stanford University, Stanford, California, United States of America; 2 Department of Chemistry, Stanford University, Stanford, California, United States of America; Griffith University, Australia

## Abstract

**Background:**

Here we continue our efforts to use methods developed in the folding mechanism community to both better understand and improve structure prediction. Our previous work demonstrated that Rosetta's coarse-grained potentials may actually impede accurate structure prediction at full-atom resolution. Based on this work we postulated that it may be time to work completely at full-atom resolution but that doing so may require more careful attention to the kinetics of convergence.

**Methodology/Principal Findings:**

To explore the possibility of working entirely at full-atom resolution, we apply enhanced sampling algorithms and the free energy theory developed in the folding mechanism community to full-atom protein structure prediction with the prominent Rosetta package. We find that Rosetta's full-atom scoring function is indeed able to recognize diverse protein native states and that there is a strong correlation between score and Cα RMSD to the native state. However, we also show that there is a huge entropic barrier to folding under this potential and the kinetics of folding are extremely slow. We then exploit this new understanding to suggest ways to improve structure prediction.

**Conclusions/Significance:**

Based on this work we hypothesize that structure prediction may be improved by taking a more physical approach, i.e. considering the nature of the model thermodynamics and kinetics which result from structure prediction simulations.

## Introduction

In 1961 Anfinsen demonstrated that the native state of a protein is encoded in its amino acid sequence and hypothesized that the native state is the lowest free energy state [1]. Since then, many researchers have dedicated their careers to understanding the driving forces underlying protein folding in order to 1) predict the native states of proteins from their amino acid sequences and 2) understand the mechanisms and pathways by which proteins fold. Collectively, these components constitute the protein folding problem [2], [3].

The protein structure prediction community has generally focused on finding a protein's native state based on its sequence. A typical approach is to develop a knowledge-based scoring function to discriminate native structures from non-native ones and to sample this potential in search of the global minimum [4]. For example, the Rosetta structure prediction package uses a Monte Carlo (MC) scheme to sample a series of scoring functions with increasing levels of chemical detail in order to identify protein native states [5]–[7]. In Rosetta and many other structure prediction schemes, the problem of finding the free energy minimum is simplified by focusing on the energetic (or score) term [8]. We note that Rosetta includes a simple implicit solvent and some implicit accounting for entropy by using information from known structures but stress that it does not explicitly account for conformational entropy. This simplification is justified by arguing that the conformational entropy of the native state is negligible and, therefore, the energetic term must be the dominant factor favoring the native state and the energy minimum should be equivalent to the free energy minimum. This approach has proved remarkably successful and has resulted in the design of a protein with a novel fold [9], accurate high-resolution structure predictions for small globular proteins [10], and the design of novel enzymes [11]. However, ignoring conformational entropy will have increasingly deleterious effects on the landscape as one moves away from the native state and this may ultimately prevent accurate structure prediction for more complex systems.

In contrast, researchers studying folding mechanisms have placed less emphasis on predicting native states and focused on understanding how proteins fold. This work is also based on potentials, or force fields. However, these potentials have been designed to reproduce our physical reality rather than to simply discriminate native and non-native protein structures. Furthermore, much emphasis has been placed on understanding the entire free energy landscape and the kinetics of traversing this landscape [2]. To accomplish these objectives numerous advanced sampling algorithms have been developed [12], as well as methods to visualize free energy landscapes [13] and determine whether or not they represent the true equilibrium distribution of the system under the given potential [14].

Here we continue our efforts to use methods developed in the folding mechanism community to both better understand and improve structure prediction. Our previous work demonstrated that Rosetta's coarse-grained potentials may actually impede accurate structure prediction at full-atom resolution [7] and this result has been confirmed by other researchers [15]. Based on this work we postulated that it may be time to work completely at full-atom resolution but that doing so may require more careful attention to the kinetics of convergence. To explore this possibility, we have used Generalized Ensemble (GE) algorithms [12] to generate projections of the landscape defined by Rosetta's full-atom scoring function. We find that these scoring functions are capable of recognizing the native states of both protein G and engrailed homeodomain, an α/β and all α-helix protein, respectively. Furthermore, the score has the desired correlation with C_α_ RMSD to the native state. However, there is a huge entropic barrier to folding and the hydrogen bonding potential does not provide any significant bias towards the native state, slowing the kinetics of convergence. Based on these insights, we believe that further advances in structure prediction may be made by taking advantage of methods and ideas developed in the folding mechanism community.

## Results and Discussion

### General Approach

**Figure 1:**
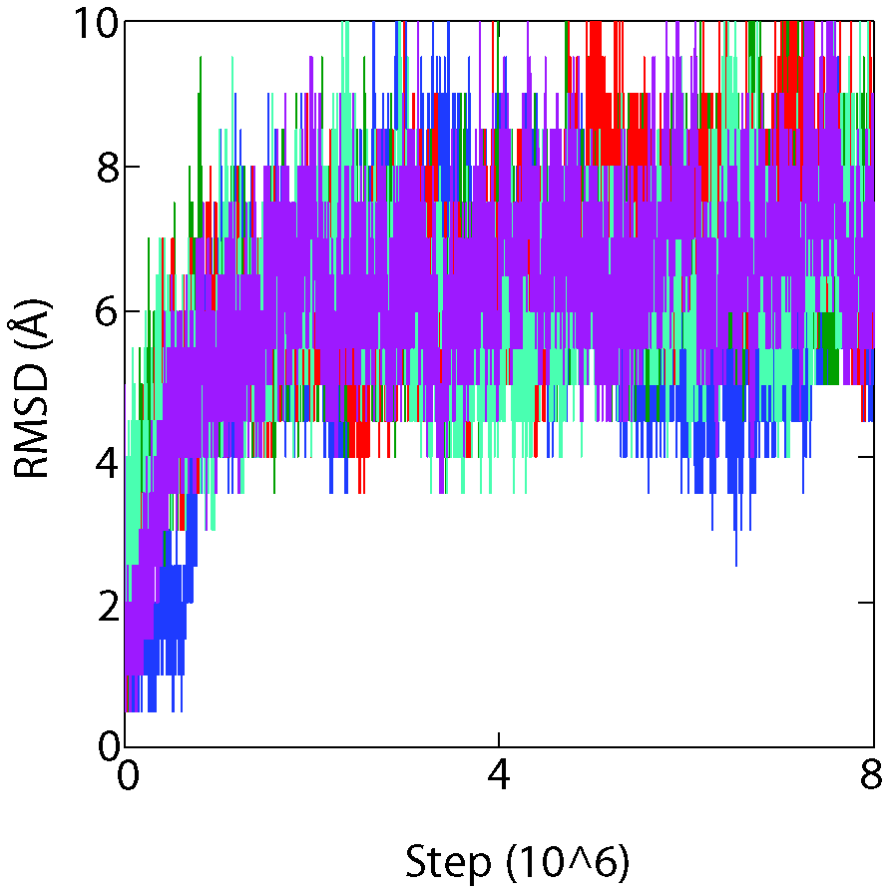
Time evolution of the C_α_ RMSD of the current umbrella center for five representative simulations demonstrating the presence of reversible folding.

In order to gain a deeper understanding of Rosetta's full-atom resolution scoring function we have implemented a variant of the Simulated Tempering (ST) algorithm [16], [17] in Rosetta. ST was originally intended to induce the system of interest to perform a random walk in temperature space so that broad sampling at high temperatures would improve mixing at lower temperatures. However, ST may be generalized to other spaces [17]. Here we define an RMSD space consisting of a number of umbrellas constraining the system to a given C_α_ RMSD from the native state. ST is then used to induce the system to perform a random walk in RMSD space without making any alterations to the temperature [18]. Furthermore, we only use MC moves rather than the combination of MC and minimization moves used in the standard Rosetta protocol. Thus, the system can move back and forth between the folded and unfolded states while remaining at equilibrium. Exchanging between umbrellas also allows the system to access all the possible conformations in a given RMSD range [19]. By performing many simulations in parallel we hope to explore all the relevant folding pathways. Figure 1 shows that this procedure results in reversible folding (i.e. multiple folding and unfolding events), confirming that our simulations have reached convergence [20]. The Multistate Bennett Acceptance Ratio (MBAR) method [21], a statistically optimal variant of the Weighted Histogram Analysis Method (WHAM) [22], is used to determine the unbiased average values of thermodynamic properties such as energies and conformational entropies as a function of the RMSD. All the thermodynamic measurements in this work are dimensionless. That is, energies and free energies are given in units of the thermal energy kT and entropies are given in units of the Boltzmann constant k.

We have applied this method to two systems: protein G (PDB code 1igd) [23] and engrailed homeodomain (PDB code 1enh) [24]. Protein G has an α/β fold while engrailed homeodomain (EH) is a 3-helix bundle. Because these systems contain both major protein secondary structure motifs our conclusions should be applicable to most protein systems.

### A Thermodynamic Perspective

**Figure 2:**
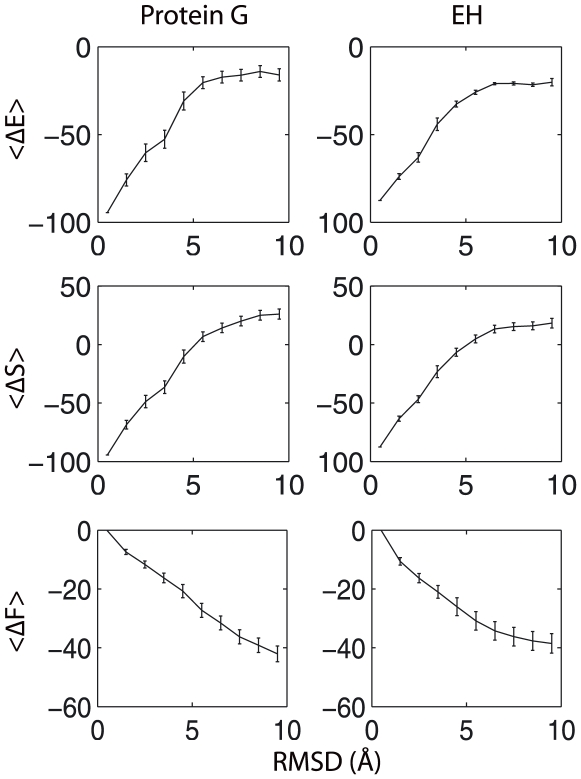
Average energy (<ΔE>), conformational entropy (<ΔS>), and free energy (<ΔF>) as a function of C_α_ RMSD for protein G and engrailed homeodomain (EH).

The average energy (or score), conformational entropy, and free energy as a function of the RMSD for both protein G and EH are shown in Figure 2. The average score has a clear correlation with the RMSD and the native state is at the scoring function's global minimum for both systems. Thus, Rosetta's full-atom scoring function is indeed able to recognize diverse protein native states. However, the conformational entropy of the native state is extremely low for both proteins. In fact, at the temperature used during full-atom Rosetta structure prediction during the CASP competitions (0.8 in arbitrary units, internal to the Rosetta code) the entropy dominates the free energy. As a result, the native state is the free energy maximum instead of the desired minimum.

This observation gives some insight into the limitations currently observed with Rosetta structure prediction. Rosetta uses a hierarchical approach in which coarse-grained structure predictions are made and then used as starting points for full-atom refinement [7]. A number of recent works have noted that for full-atom refinement to be successful, i.e. reach RMSD values less than 2 Å, the initial configuration must be within a “radius of convergence” of about 3 Å from the native state [6], [8]. Our results show that the free energy difference between 3 Å and 2 Å is about 5 kT and, therefore, sampling a 2 Å structure when starting from a 3 Å structure is extremely unlikely. The improbability of moving to lower RMSD structures is consistent with the fact that one to ten thousand independent runs must be performed in order to find a few accurate full-atom structures with Rosetta's *ab initio* structure prediction protocol [10].

### Temperature Dependence of the Free Energy

The relative importance of the energetic and entropic contributions to the free energy may be tuned by adjusting the temperature (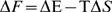). Namely, the energetic term will dominate at sufficiently low temperatures while the entropic term will dominate at higher temperatures. By assuming that the average energy and conformational entropy are independent of temperature we are able to predict the temperature dependence of the free energy. We can then predict what temperature one would have to use in Rosetta structure prediction in order for the free energy landscape to have the desired correlation with the RMSD.

**Figure 3:**
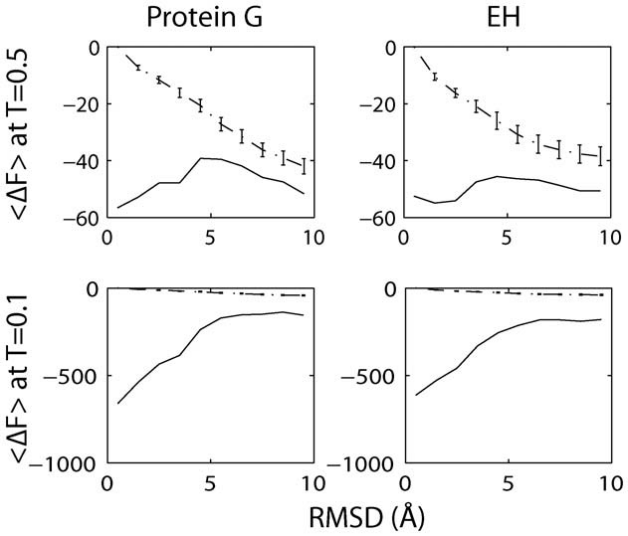
Average free energies (<ΔF>) as a function of C_α_ RMSD for temperatures of 0.5 and 0.1 for protein G and engrailed homeodomain (EH). The black lines are the hypothesized free energy at the given temperature and the dash-dot lines are the free energy at temperature 0.8 shown for reference.

We find that the free energy landscape has the desired shape (i.e. stable native state, unstable unfolded state) at temperatures below 0.5, as shown in Figure 3. At temperatures above 0.5 the free energy landscape still has a maximum at the native state. At a temperature of about 0.5 there are still non-trivial barriers between the native and unfolded state but the free energy landscape is essentially flat relative to other temperatures.

### Exploiting the Temperature Dependence

While the projections of the thermodynamic landscapes shown in Figures 2 and 3 appear to be smooth, the true landscapes are actually quite rugged due to energetic terms like hydrogen bonding and Van der Waals interactions. In order to explore this space the standard Rosetta full-atom refinement protocol uses a combination of MC and minimization moves [7]. The minimization moves are intended to guide the protein towards the native state at the energy minimum while the MC moves are intended to help the protein overcome small barriers. For the MC moves to perform this function they must use a sufficiently high temperature to overcome small barriers but a low enough temperature to avoid mitigating the effectiveness of the minimization moves. Simply running the standard protocol at a lower temperature is likely to destroy this balance and prevent the system from overcoming even trivially small barriers, thus drastically slowing the dynamics. However, using our insights into the temperature dependence of the free energy landscape it may be possible to devise a temperature ST protocol that could overcome this roughness and reach the native state.

To test this hypothesis we have implemented a temperature ST version of the full-atom Rosetta refinement protocol, as well as a variant of the standard protocol that runs at a temperature of 0.1. For the ST variant we used a temperature range of 0.1 to 0.5 and a purely MC move set in order to obey detailed balance. Broad sampling should be possible at a temperature of 0.5 because of the relative flatness of the landscape, while at lower temperatures the native state should be favored. Temperatures above 0.5 are not used because they would favor unfolding. The low temperature variant allows us to ensure that any improvements seen with the ST variant over the standard protocol are not simply the result of running at lower temperatures. Both the standard and low temperature variants use the full set of MC and minimization moves available in Rosetta.

**Figure 4:**
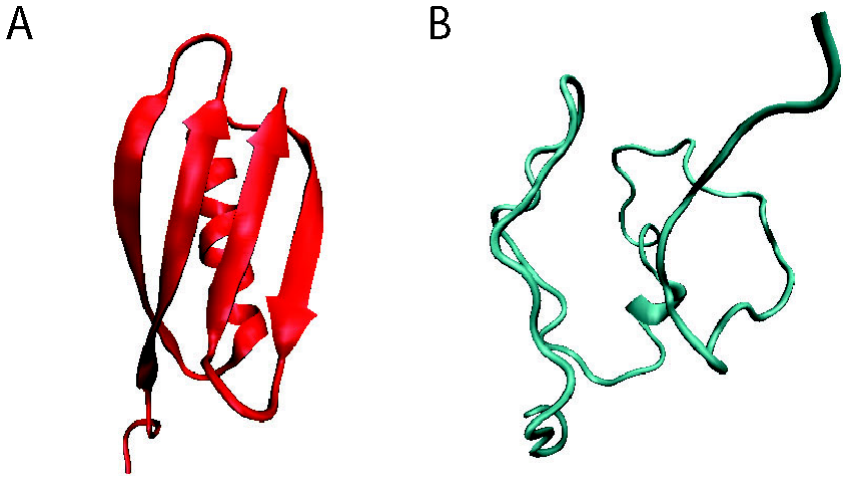
(A) The native structure of protein G and (B) the 5.7 Å starting structure used for comparing the ST and Standard Rosetta variants.

**Figure 5:**
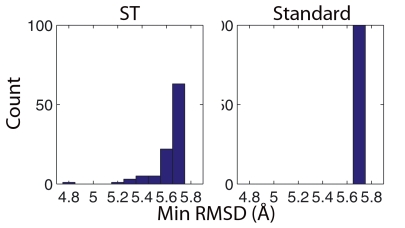
Distribution of the minimum C_α_ RMSD values reached by 100 Simulated Tempering (ST) and 100 standard Rosetta runs started from a 5.7 Å structure. Results for both the low temperature and standard Rosetta variants were identical so only a single plot is shown.

Our ST variant is found to outperform both standard Rosetta and the low temperature variant. For each of these three protocols we performed 100 runs starting from a 5.7 Å structure, well beyond the radius of convergence, drawn from our umbrella sampling simulations. Figure 4 shows our 5.7 Å starting structure alongside protein G's native state as a reference. Figure 5 shows histograms of the lowest RMSD found in each run. One ST run reached an RMSD value of 4.8 Å and 37% of the ST runs found structures with RMSD values lower than the initial configuration. However, neither the standard protocol nor the low temperature variant were able to find any structures with RMSD values less than that of the initial configuration. The increased ability of our ST protocol to move towards the native state demonstrates that utilizing explicit knowledge of the entropic contribution to the free energy may improve structure prediction, even when the physical conformational entropy is not of interest.

### Physical Perspective on Energetic Terms

A physical perspective may also be taken in order to evaluate and improve individual energetic terms. For example, Rosetta's hydrogen bonding term [25] is seen as a critical component of the full-atom scoring function [8]. While this term agrees with quantum calculations [26], it has been found empirically that the hydrogen bonding potential only helps discriminate between models within about 3 Å of the native state [25].

**Figure 6:**
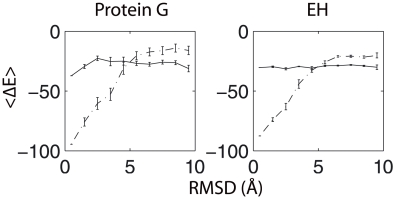
Relative magnitude of the average hydrogen bonding energy (solid line) versus the total average energy (dash-dot line) as a function of Cα RMSD for protein G and engrailed homeodomain (EH).

We find that the hydrogen bonding term actually impedes the kinetics of convergence while providing only a minor energetic advantage to near-native states and, therefore, ultimately impedes rapid and accurate structure prediction. Figure 6 shows that the average hydrogen bonding energy is somewhat lower within about 3 Å of the native state for protein G but not for EH. For both systems, however, the average hydrogen bonding energy is basically flat relative to the total energy. Because the average hydrogen bonding energy is flat, it does not necessarily provide any guiding force to bias the system towards the native state.

Shmygelska and Levitt have reported that Rosetta's hydrogen bonding potential is better able to discriminate native from non-native states than the low-resolution potentials [15]. The most likely explanation for this apparent discrepancy is that they weighted the hydrogen bonding term more heavily. During our simulations the long-range hydrogen bonding term was weighted by a factor of one while the short-range term was weighted by a factor of 0.5, following the protocol used by the Baker group in CASP 7. If these terms were weighted more heavily relative to the rest of the potential a stronger bias towards the native state could arise. For example, the small dip we observe in the hydrogen bonding term for protein G could become quite substantial. Comparing our results with those of Shmygelska and Levitt is also complicated by the fact that they sampled the hydrogen bonding term in the context of Rosetta's less accurate low-resolution potentials while we have sampled it in the context of the more accurate full-atom potential. A more extensive comparison of our methods in the context of the full-atom potential is an interesting future direction.

We suggest that structure prediction potentials could possibly be improved by avoiding such flat terms or reweighting them such that they provide a substantial biasing force towards the native state. We note that proteins can have surprisingly fast kinetics, with some small proteins folding on the microsecond time scale [27]. One outstanding question is whether it is even feasible to design a knowledge based potential that can accurately identify protein native states and have kinetics that are faster than physical kinetics. If not, physics based methods may actually be the fastest algorithms for complex systems as they may be able to take advantage of the evolutionary optimization or the physical processes for kinetics present in the natural kinetics of protein folding. Even if this is not the case, our results show that structure prediction may benefit by taking advantage of ideas developed to better understand folding mechanisms. Informatics approaches that incorporate more physical insights into protein folding mechanisms are thus an interesting direction [28]–[30].

### Conclusions

Our results demonstrate that explicitly accounting for conformational entropy and considering the kinetics of convergence may improve structure prediction even if physical conformational entropies and kinetics are not of interest. For example, by understanding the interplay between energy and conformational entropy one can choose an optimal temperature or set of temperatures to use for exploring conformational space. By considering the kinetics of convergence one can ensure that this space can be explored rapidly, resulting in computationally efficient structure prediction protocols. An outstanding question is whether it is possible to design knowledge-based potentials with better entropic and kinetic properties than our physical reality. If not, physics based structure prediction may ultimately be necessary for more complex systems. Whether or not this is the case, our results show that structure prediction may benefit by taking advantage of ideas developed to better understand folding mechanisms.

## Materials and Methods

All structural representations were generated using VMD [31].

### Temperature ST

Temperature ST [16], [17] simulations perform a random walk within a pre-determined temperature set {T_n_}. This is accomplished using an expanded Hamiltonian
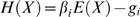
where 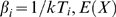 is the energy (or score) of the current configuration (X), and g_i_ is the weight corresponding to T_i_. At regular intervals the simulation attempts to move either up or down in temperature space with equal probability. The probability of accepting a given move is
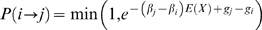
where P(i→j) is the probability of moving from T_i_ to T_j_.

Our temperature ST simulations used a temperature list of 0.1, 0.15, 0.2, 0.3, 0.4, and 0.5 in arbitrary units internal to the Rosetta code and temperature exchanges were attempted every 50 steps. All weights were determined using the Simulated Tempering Equal Acceptance Ratio (STEAR) method [7]. This method obtains an initial estimate of the weights from short constant temperature simulations at each temperature and then refines these weights in subsequent ST simulations before holding them constant in the final data collection phase. Two iterations of weight refinement consisting of 100 runs of 600,000 steps were performed for temperature ST simulations, followed by 100 runs of 600,000 steps for data collection. In order to maintain detailed balance the ST simulations only used MC moves in torsion space.

### RMSD ST

RMSD ST simulations perform a random walk amongst a predetermined set of umbrellas constraining the system to a given RMSD from the native state without changing the system's temperature. In this case the expanded Hamiltonian and probability of accepting a move are
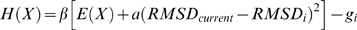


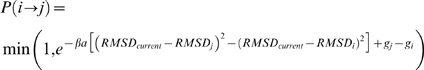
where 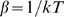, E(X) is the energy of the current configuration (X), RMSD_current_ is the current RMSD from the native state, RMSD_i_ is the center of umbrella i, and “a” determines the strength of the spring constraining the system to a given umbrella.

Our RMSD ST simulations used umbrellas centered at RMSD values from 0.5 to 10 Å at 0.5 Å intervals and jumps between neighboring umbrellas were attempted every 50 steps. The “a” parameter was set to three. All weights were determined using the Simulated Tempering Equal Acceptance Ratio (STEAR) method [7]. This method obtains an initial estimate of the weights from short umbrella simulations at each umbrella (without any jumps between umbrellas) and then refines these weights in subsequent RMSD ST simulations before holding them constant in the final data collection phase. Three iterations of weight refinement consisting of 100 runs of 1,700,000 steps were performed for RMSD ST simulations, followed by 100 runs of 900,000,000 steps for data collection. In order to maintain detailed balance the RMSD ST simulations only used MC moves in torsion space.

### Rosetta

For an overview of the Rosetta structure prediction algorithm and the command-line options used in this study see reference [7]. The full Rosetta move set was used for standard Rosetta runs. The same number of moves was used when comparing standard Rosetta runs with ST.
